# Nucleotide Dependent Switching in Rho GTPase: Conformational Heterogeneity and Competing Molecular Interactions

**DOI:** 10.1038/srep45829

**Published:** 2017-04-04

**Authors:** Amit Kumawat, Suman Chakrabarty, Kiran Kulkarni

**Affiliations:** 1 Physical and Materials Chemistry Division, CSIR-National Chemical Laboratory, Pune 411008, India; 2 Division of Biochemical Sciences, CSIR-National Chemical Laboratory, Pune 411008, India

## Abstract

Ras superfamily of GTPases regulate myriad cellular processes through a conserved nucleotide (GTP/GDP) dependent switching mechanism. Unlike Ras family of GTPases, for the Rho GTPases, there is no clear evidence for the existence of “sub-states” such as state 1 & state 2 in the GTP bound form. To explore the nucleotide dependent conformational space of the Switch I loop and also to look for existence of state 1 like conformations in Rho GTPases, atomistic molecular dynamics and metadynamics simulations on RhoA were performed. These studies demonstrate that both the nucleotide-free state and the GDP bound “OFF” state have very similar conformations, whereas the GTP bound “ON” state has unique conformations with signatures of two intermediate states. The conformational free energy landscape for these systems suggests the presence of multiple intermediate states. Interestingly, the energetic penalty of exposing the non-polar residues in the GTP bound form is counter balanced by the favourable hydrogen bonded interactions between the γ-phosphate group of GTP with the highly conserved Tyr34 and Thr37 residues. These competing molecular interactions lead to a tuneable energy landscape of the Switch I conformation, which can undergo significant changes based on the local environment including changes upon binding to effectors.

Rho GTPases are small (~20 kDa), monomeric, Guanine nucleotide binding proteins that belong to the RAS superfamily. These proteins regulate a variety of cellular processes such as cytoskeletal organisation, cell cycle progression, cell migration and gene transcription by acting as bio-molecular switches^1^,^2^,^3^,^4^. The highly conserved switching mechanism involves shuttling of Rho GTPases between their GTP bound (ON) active state and GDP bound (OFF) inactive state^5^. The transition of Rho GTPases between their ON and OFF states is regulated by GEFs (guanine nucleotide exchange factors) and GAPs (GTPases activating proteins)^6^. GEFs catalyse the activation of Rho GTPases by exchanging their bound GDP with GTP, whereas GAPs inactivate them by promoting their intrinsic GTP hydrolysing capacity. Guanine nucleotide dissociation inhibitors (GDIs) provide additional regulation by sequestering the GDP bound form to maintain adequate pool of inactive Rho GTPases in the cytosol^7^. Aberrations in the functional Rho GTPases, mainly in their switching action, have been implicated in a number of diseases like cancers, developmental disorders and bacterial infections, where they occur in constitutively active state^8^,^9^,^10^,^11^,^12^.

Several structural studies on small GTPases have shown that two divergent regions in these proteins, called switch I (SWI, residues 28 to 40 in RhoA) and switch II (SWII, residues 61 to 81) regions play critical role in exerting biological functions of these proteins^13^,^14^,^15^,^16^,^17^,^18^. These two regions not only constitute the nucleotide binding pocket but also engage with their regulators (GEFs and GAPs) and effectors (like kinases)^12^,^19^. These loops also perch the Mg^2+^ binding site, essential for functioning of the protein. In the ON state, the γ-phosphate of the bound GTP forms two hydrogen bonds between the side-chain oxygen of SWI Thr37 and the main-chain oxygen of SWII Gly62 according to RhoA numbering (Thr35 and Gly60 according to H-Ras numbering, respectively). The change in structure upon the loss of the γ-phosphate, on GTP hydrolysis, is termed as the loaded-spring mechanism^5^. This change defines the activation and inactivation mechanism of the GTPases.

Crystal structures of both GTP and GDP bound states of Rho GTPase exhibit multiple SWI conformations, suggesting plausibility of an ensemble of “micro” ON and OFF states. Interestingly, NMR studies on H-Ras, a member from the RAS superfamily, have revealed that SWI region of the GTP bound state has multiple interconvertible conformations, whereas no such conformational fluctuations are seen in the GDP bound state^20^. Based on these experimental studies the GTP bound state for Ras GTPase has been classified into two states, known as state 1 (inactive) and state 2 (active)^21^,^22^. Modest alterations in the coordination between Thr35 and Mg^2+^ are shown to alter these states of the Ras GTPase. Furthermore, state 2, which is most prevalently seen in the crystal structures of the wild type Ras, represents the high affinity state for the effectors. On other hand state 1 represents a different (GDP-like) state of the protein with a substantially reduced affinity for effectors, often seen in the crystal structures of mutant of Ras (T35S)^23^. Conformational fluctuations of these two states for Ras GTPases have been extensively studied using normal mode analysis and molecular dynamic simulations^24^,^25^,^26^,^27^,^28^,^29^,^30^,^31^,^32^. The minimum energy pathway between the two states has been analysed with the conjugate peak refinement method^33^,^34^. However, there is very limited information on the conformational states of Rho GTPases. Experimental studies on Cdc42, a Rho GTPase, have shown the protein to exist only in the state 2 conformation^35^. It is interesting to note that, despite sharing significant structure and sequence homology with H-Ras and having the conserved threonine in the SWI region, Cdc42 predominantly exists in the state 2 conformation.

Therefore, to explore the conformational states of SWI region of Rho GTPases we have performed extensive molecular dynamics (MD) simulations on RhoA, a *bonafide* member of Rho Family of GTPases. Availability of several crystal structures of RhoA in different forms made this protein an amiable model system to investigate the conformational states. To further understand the conformational features, free energy calculations were performed. These studies indicate plausibility of existence of state 1 like conformation of GTP bound Rho GTPases. A comparative analysis of the structural signatures of SWI conformations, corresponding to the free energy minima, provides a qualitative explanation for the sparse occurrence of state 1 conformation in Rho GTPases. Finally, analysis of conformation of SWI residues in the GTP bound state reveal substantial solvent exposure of the hydrophobic residues, where the unfavourable solvation energy gets overcompensated by the favourable electrostatic interaction with the nucleotide (GTP). Perhaps, this unique conformational state with hydrophobic exposure has an important role in effector recognition.

## Results and Discussion

### MD simulations of GDP, GTP and nucleotide free state of RhoA

**Figure 1:**
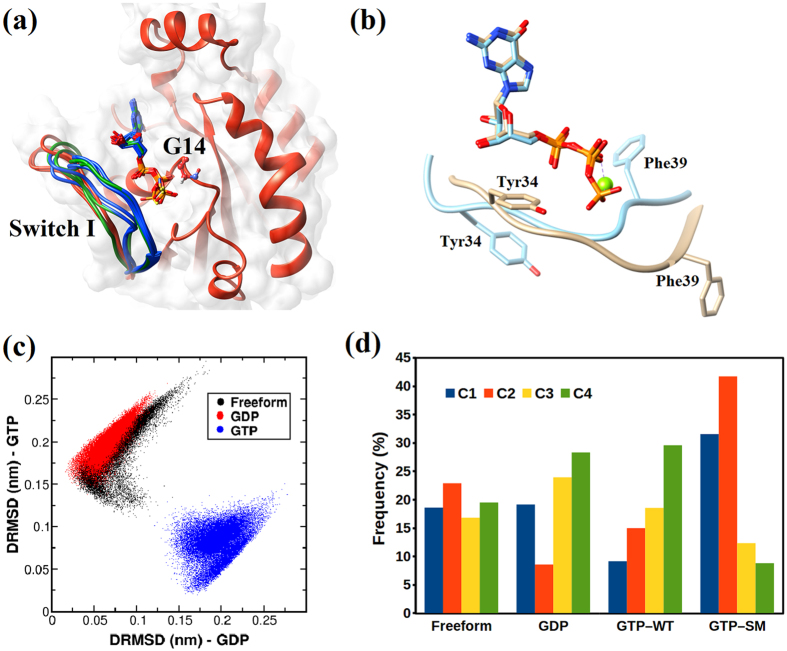
(**a**) Superimposition of the Switch I loop using the available crystal structures of RhoA. The conformational preference of the loop region has been indicated by the following colors: nucleotide free (red), GTP bound (blue) and GDP bound (green). (**b**) Open and closed conformation in GDP and GTP bound form exhibited by change in the orientation of Tyr34 and Phe39 (**c**) Distance based RMSD values (DRMSD) for the trajectories: free form (black), GDP bound (red) and GTP bound (blue). The coordinates are obtained as the DRMSD values of each structure calculated with respect to both the GTP bound structure (Y axis) and the GDP bound structure (X axis) in order to demonstrate the structural distribution and similarities. (**d**) Population Analysis using K-means Clustering.

To address the conformational heterogeneity in the Rho GTPases, we have superimposed the available crystal structures of RhoA in the nucleotide free form, the GDP bound and the GTP bound form (Fig. 1a). This analysis shows that the crystal structures of Rho GTPases in GDP bound and GTP bound form show overall close structural similarity. However, SWI and SWII regions, particularly side chains of Tyr34 and Phe39, seem to exhibit largest deviation among crystal structures (Fig. 1b). Although structurally diverse, the SWI and SWII regions exhibit consensus sequence, which might play an important role in effector binding. To further explore the nucleotide dependent conformational signatures in Rho GTPases and possible existence of intermediate states, we performed 2 μs of atomistic MD simulations on RhoA in its nucleotide free, GDP bound, GTP bound and the GTP bound G14V mutated form. Extensive structural and thermodynamic characterisation of the conformational ensemble observed in the MD simulation is reported in the following sections. We have calculated the RMSD of the backbone atoms with respect to the crystal structures to monitor convergence and stability of the protein (Supplementary Fig. S1a). Although the times scales of MD simulation limit to track the complete transition of the protein between the active and inactive states, shifting of Tyr34 in open and closed conformations, which Rho GTPases exhibit in their GDP and GTP bound state, were observed (Fig. S2).

The RMSF profile of residue-wise fluctuations shows larger fluctuations in the SWI and SWII regions as compared to the other regions of the protein (Fig. S1b). However, both the loop regions show significantly lower fluctuations upon nucleotide binding as compared to the nucleotide free state, which signifies the role of these regions in nucleotide binding and/or effector recognition. Earlier simulation studies have shown that mutations in the p-loop or switch regions can significantly alter the degree of flexibility of these regions^36^,^37^,^38^,^39^. A point mutation (P29V) locks the RAC1 in the GTP bound activated state, accompanied by the enhanced flexibility in the SWI region and rigidity in the SWII region^36^. However, this work would retain focus on the conformational heterogeneity in the SWI loop, since the conformational signature of this region are employed to define substates like “state 1” and “state 2”^21^,^22^,^23^.

### Identification of conformational states of SWI region in Rho GTPases

To assess the conformational spread of SWI region from the GTP bound and GDP bound states, distance based RMSD (DRMSD) has been used as a metric (see Methods section for details). Due to the inherent degeneracy of RMSD-like quantities, a single reference structure is not enough to characterize the structures when the deviation between the reference structure and structures under query are large. Thus, we have used two different references structures (crystal structures for GDP-bound inactive state and GTP-bound active state) to achieve better resolution in classifying the MD trajectories. Fig. 1c demonstrates the spread of conformational states sampled in the MD simulations of the respective systems. Lower *X* values (DRMSD w.r.t GDP-bound inactive state) and *Y* values (DRMSD w.r.t GTP-bound active state) indicate higher similarity to GDP and GTP bound structures, respectively. The distribution clearly indicates that the SWI region in the nucleotide freeform and GDP bound inactive state has very similar conformations, whereas the GTP bound active state has very unique structures corresponding to an isolated domain in this conformational landscape. Interestingly, there is a subtle tendency in the freeform to approach the GTP bound states, which will be further demonstrated through free energy calculations in a later section.

Although DRMSD could quantify the similarity/dissimilarity with reference structures, it provides a coarsened global picture of the structural differences. In order to achieve a more microscopic conformational clustering, such as state 1 and state 2 of the ‘ON’ form (GTP bound form), of the observed MD trajectories an unsupervised machine learning approach was used. We have employed the k-means clustering method^40^,^41^,^42^,^43^,^44^,^45^. A coarse representation of different clusters (Fig. 1d) reveals that the GTP bound conformational states of the SWI loop region form two distinct clusters (C3 and C4), whereas the conformational states of nucleotide freeform and GDP bound states show considerable similarity, as observed in case of DRMSD analysis.

To further investigate the effect of mutation on the conformational states of SWI, the procedure was repeated for the simulation results of single mutant (G14V) RhoA-GTP, which is observed in myriad type of cancers^46^,^47^. Equivalent mutants in H-Ras have shown SWI region of this protein to predominantly adopt the state 1 (inactive GDP bound) like conformation^48^. Hence the objective here was to test whether RhoA has similar tendencies. The results obtained from k-means clustering with the feature set from single mutant RhoA-GTP showed clustering pattern of forming two distinct clusters, as observed for the Wild Type RhoA-GTP structures. However the occurrence of one of the state (C2) seems more dominant. This analysis clearly demonstrates that GTP bound form of RhoA is capable of adopting two preferential states that might be analogous to the state 1 and state 2 of the GTP bound H-Ras. On the contrary, nucleotide free form and GDP bound form of RhoA do not exhibit preferential conformational states. It is interesting to note that the demarcation of the preferences for the states is more illustrious for the mutant, similar to what has been observed for H-Ras. In case of H-Ras this mutation drives the GTP bound protein towards the inactive, state 1 conformation. However, for RhoA this mutation impede the protein from binding to nucleotides and effectors^49^.

### Conformational free energy landscape of Switch I region

In case of H-Ras, several MD simulations have been performed to delineate state 1 and state 2 conformations of the protein. In most of these studies the distance between Thr35 and the γ-phosphate of the bound GTP, coordination between Thr35 and Mg^2+^ ion and the H-bond between Gly60 and γ-phosphate are considered as an important structural indicator of the states^26^,^50^,^51^. To our knowledge, there are no crystal structures of Rho GTPases attributed to these particular conformational states of the SWI loop in the GTP bound form. Hence to identify the structural signatures of state 1 and state 2 like conformations in Rho GTPases, there was a need to explore a large conformational space of SWI region and sample the conformations based on their free energy to identify the energetically stable and metastable states in terms of known active and inactive states.

**Figure 2:**
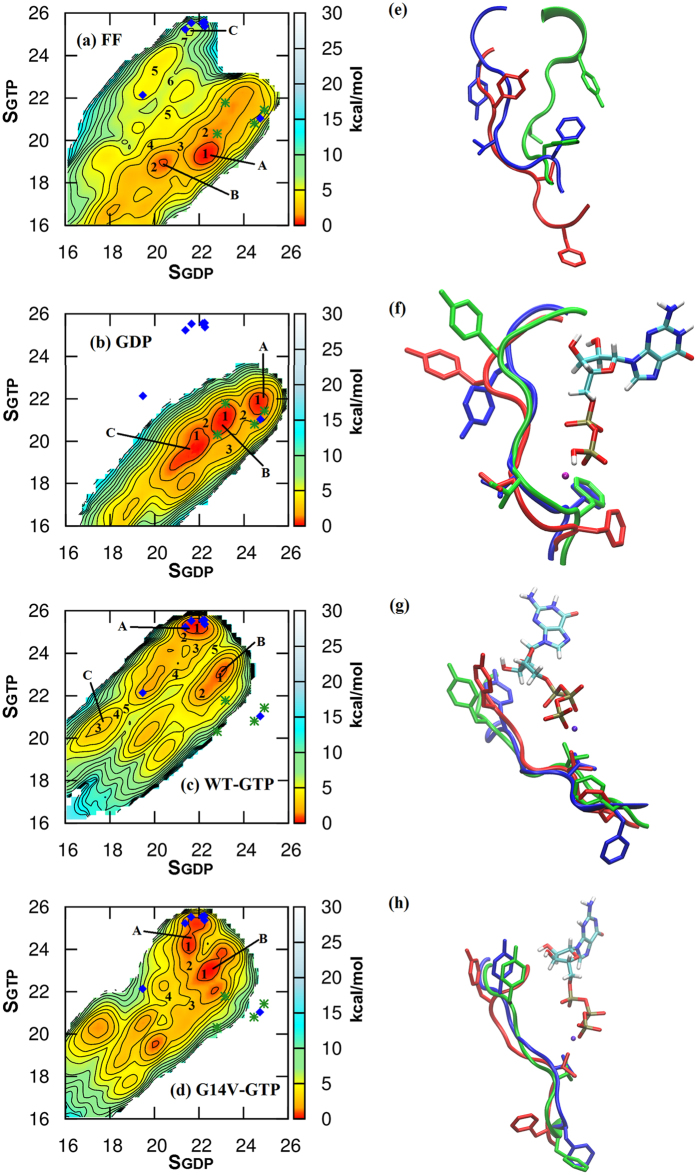
Free energy surfaces corresponding to (**a**) Freeform, (**b**) GDP bound form, (**c**) Wild type (WT) GTP bound form, and (**d**) GTP bound form with G14V mutation on the left panel. The position of the few representative available crystal structures have been marked on the free energy surface with the following colors: GDP (green star) and GTP (blue diamond). On the right panel (**e**–**h**), Structural superimpositions of switch I region conformations for different minima labeled as A(Blue), B(Green), C(Red).

Similar to the unsupervised clustering, the free energy surface incorporates the information about the possible conformational ensemble describing the energetically stable and metastable states. Although unbiased MD simulations provide very useful molecular insights regarding the dynamics of biomolecules, such simulations may have limited capabilities in sampling a complex free energy landscape. In particular, large-scale conformational changes in biomolecules are often associated with high free energy barriers (greater than a few k_B_T) making them inaccessible (or poorly sampled) to normal MD simulations. Hence the well-tempered metadynamics method has been used to accelerate the conformational sampling of the SWI region in each of the systems under different consideration, namely (i) the GDP bound form, (ii) the GTP bound form, (iii) the nucleotide freeform, and (iv) the GTP bound form of the G14V mutation, which is known to exist in a constitutively active state. The free energy surfaces (FES) computed using the reweighting approach^52^ have been shown in Fig. 2.

To identify the ‘active’ and the ‘inactive’ conformational states of the SWI loop “dihedral similarity” (*S*) collective variable was chosen (See methods section). The two collective variables *S*_*GDP*_ (X axis) and *S*_*GTP*_ (Y axis) are the *S* parameters calculated with respect to the GDP bound and GTP bound reference structures, respectively, that indicate the dihedral similarity of SWI region. According to the definition of the *S* parameter, larger *S* values would indicate higher similarity with the reference structure, e.g. structures with higher *S*_*GDP*_ values will have greater degree of similarity to the reference GDP bound structure. The two dimensional space shown in Fig. 2 (*S*_*GDP*_, *S*_*GTP*_) is the reduced representation of the full conformational space of the backbone (2 ^N^ for the φ/ψ angles of N residues). In order to validate this representation, the (*S*_*GDP*_, *S*_*GTP*_) values for the SWI loop of total 36 crystal structures of RhoA, Rac1 and cdc42 GTPases in their GDP bound and GTP bound form (Supplementary Table S1) were mapped on each of these free energy surfaces. In Fig. 2, only crystal structures existing in *apo* nucleotide bound state have been superimposed. Interestingly the (*S*_*GDP*_, *S*_*GTP*_) collective variables corresponding to the majority of GTP and GDP bound crystal structures form clusters, which nicely overlap with the global minima in the FES of the corresponding system. This clearly indicates that the conformational states discovered by the metadynamics sampling closely resembles the available structural data. Also, it is clear from the representation that the (*S*_*GDP*_, *S*_*GTP*_) collective variables have the ability to distinguish between the active and inactive conformational states.

A comparative analysis of these maps suggests that, in the absence of any nucleotide (Fig. 2a), a large number of conformational states are thermally accessible as indicated by the presence of multiple stable states in the dihedral conformational space. This unveils the highly flexible nature of the loop in the absence of nucleotide (GDP/GTP). On the other hand the GDP bound structure is characterised with much more structured free energy surface with lower number of thermally accessible conformational states compared to the freeform. The FES for the GDP bound form (Fig. 2b) has three minima separated by rather small (~2 kcal/mol) barrier (Table S2) indicating high degree of flexibility between these states. However, the GTP bound conformations are never visited in a GDP bound state, which would have required to cross much higher free energy barrier (>15 kcal/mol).

However, in case of GTP bound structure the number of deep minima (stable states) is even lower (Fig. 2c). This signifies the presence of a few distinct conformational states in both nucleotide bound systems unlike the freeform. But the position of the minima in the GTP bound state is significantly different as compared to the GDP bound system, indicating the unique conformational signatures of the GTP bound ON state. Interestingly, for the GTP bound form other metastable intermediate states, e.g. the state marked “B” with slightly higher free energy (~0.7 kcal/mol) than the state “A”, were observed. However, the barrier of the interconversion (A to B) is 5 kcal/mol.

In a previous study by Gorfe *et al*.^25^, it has been shown that the Ras mutants occur in the intermediate region between the GTP bound active and GDP bound inactive states and hence they have hypothesized the existence of a lower free energy barrier in the oncogenic variants. In the case of RhoA the oncogenic mutation G14V is known to be in a constitutively active state leading to an uncontrolled growth^47^,^53^. To investigate the effect this mutation on the conformational preference of the SWI loop of RhoA, FES for the G14V mutation in the GTP bound state was calculated (Fig. 2d). Although, the qualitative nature of the FES remains similar to the wild-type GTP bound form (Fig. 2c), but in the G14V system the identity of the global minimum shifts to an intermediate state (marked “B”). The ∆G for transition from A to B is +0.7 kcal/mol and −0.4 kcal/mol for the WT and G14V, respectively. Furthermore, the barrier of transition between the GTP bound active and GDP bound inactive states is lowered by 3 kcal/mol, thus signifying enhanced flexibility of the loop. It has been shown earlier that the G14V mutant becomes constitutively active since the chemical step of deactivation (GTP hydrolysis) is hindered in this mutant^54^. Our results for the G14V mutation corroborates the decreased free energy barrier with the increased flexibility in the switch I region as observed in the earlier study^36^. In addition, the current results indicate that the loop region undergoes a population shift of the conformational states in addition to increased flexibility, which might promote binding to any arbitrary effector proteins through induced fit mechanism. Thus, the G14V mutant might be able to activate a large variety of downstream effector with lower degree of selectivity/specificity.

### Rho GTPases exhibit signatures of state 1 and state 2 in their GTP bound form

For H-Ras, the state 1 and state 2 conformations of the GTP bound protein was defined in terms of the distance between Thr35 and the γ-phosphate of the bound GTP. However, dynamics of other neighbouring residues, such as Tyr32 in H-ras (Tyr34 in RhoA numbering), were also shown to contribute in stabilizing and defining these states^55^. For Rho GTPases there is no structural data attributing their state 1 and state 2 conformations. Hence all available GTP and GDP bound structures of the GTPases belonging to this family were compared to define the “active” and “inactive” states of the protein. The only difference expected in the GTP bound forms, compared to the GDP bound forms, is stabilization of the SWI region by the Thr37 and γ-phosphate mediated interactions. However, for Rho GTPases, in addition to this interaction there is a correlated motion of Tyr34 and Phe39. In the GDP bound form the side chain of Tyr34 points outward; whereas it flips inside to form hydrogen bond with the γ-phosphate in case of the GTP bound state (Fig. S3a). Similarly, the hydrophobic Phe39 side chain is buried in the GDP form, whereas it flips out in the active state to become solvent exposed (Fig. S3a)^56^. We have identified the nucleotide dependent correlation between the orientation of Tyr34 and Phe39 based on the distance between the nearest interacting partner with respective side-chain in the unbound and bound state (Fig. S3b). This was further substantiated with the analysis of average number of water molecules around the Tyr34 and Phe39 side-chains (Fig. S3c). Since these particular residues, Tyr34 and Phe39 and their correlated motion is uniquely found in Rho GTPases (Fig. S2), conformations akin to these were used as structural signature to define the active and the inactive state of the GTPase. To validate this assignment, conformations of SWI regions of the trajectories corresponding to the three minima in the FES of the nucleotide freeform, GDP bound form, GTP bound form and GTP bound form of the G14V mutation were carefully examined (Fig. 2e–h). The minima corresponding to the nucleotide free form do not show any correlated conformations of Try34 and Phe39, indicating absence of well-defined states of the GTPase in this form. However, the minima corresponding to the GDP bound form have similar conformations of Phe39 and a modest change in the open conformation of the Tyr34. Perhaps, they all correspond to the same GDP bound state with marginal deviations. Interestingly, for the GTP bound form based on the conformations of the SWI region, the minima can be grouped into two distinct classes: one with Phe39 as in the active state (blue) and the other class related to the energy minima “B” & “C”, belonging to an intermediate state. In these two minima the Try34 has the active state conformation. Perhaps, these two classes represent state 2 (corresponding to “A”) and state 1 (corresponding to “B” or “C”) of the GTP bound Rho GTPases, equivalent to those observed for the Ras family of GTPases. In case of GTP bound-G14V mutant, which is known to drive the protein towards state 1 (inactive), all the minima have Phe39 in an intermediate conformation similar to the one designated as the WT-GTP state 1. Thus, this particular observation further corroborates the existence of state 1 and state 2 like conformations in Rho GTPases. In Fig. S2, we demonstrate the probability distribution of the minimum distance between Tyr34 and GTP/GDP highlighting the multiple possible intermediate orientations of Tyr34 depending on the nucleotide binding state. The probability distribution of the χ dihedral angle of Tyr34 (Fig. S4) also clearly demonstrates the dramatic population shift in a nucleotide dependent manner as well as the G14V point mutation.

### Competing molecular interactions leading to conformational selectivity and correlated motion

**Figure 3:**
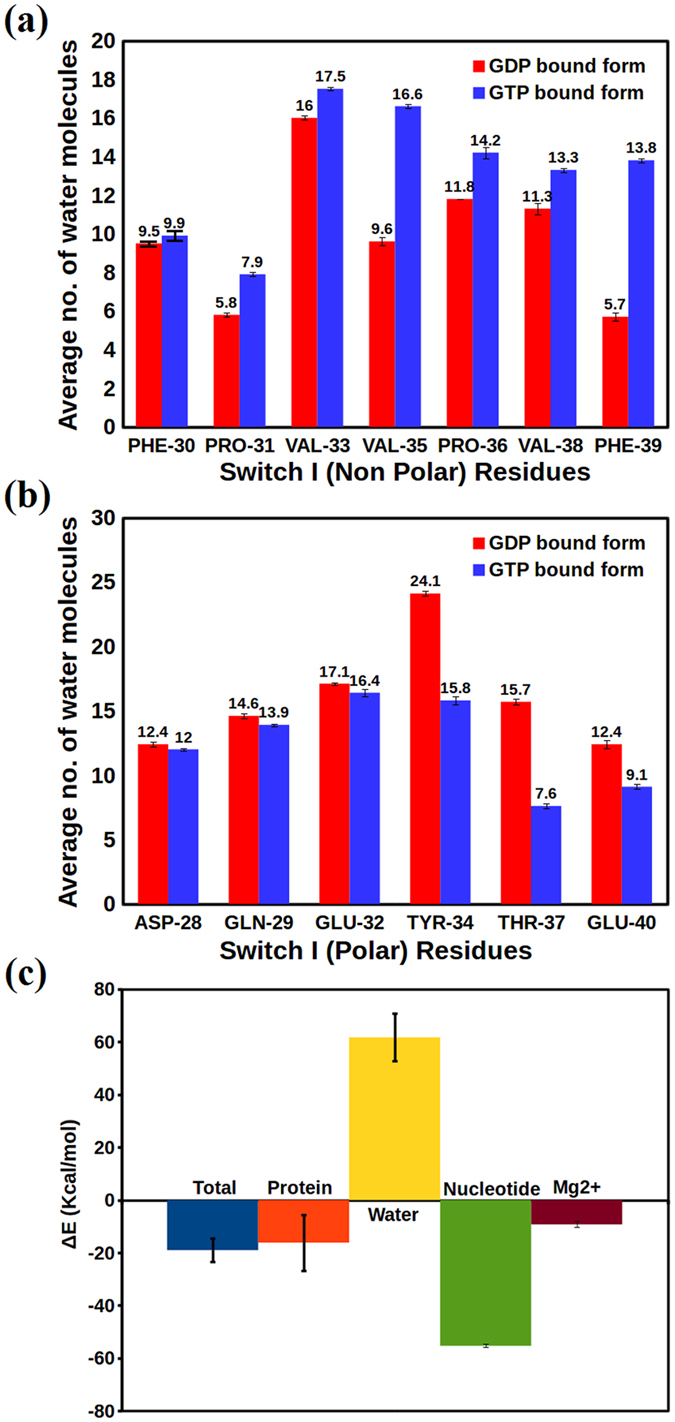
Comparison of the average number of water molecules around (**a**) non-polar residues and (**b**) polar residues. The red and blue bars indicate the GDP bound and GTP bound states, respectively. (**c**) The stabilization energy of the GTP bound state with respect to the GDP bound conformational state, i.e. Δ*E* =*E*_*GTP*_−*E*_*GDP*_, In addition to the total stabilization energy (blue), we have also shown the various components due to protein (orange), water (yellow), nucleotide (green) and the Mg^2+^ ion (brown).

To further understand the molecular interactions that stabilize the respective active and inactive conformational states of the Rho GTPases the role of solvation was investigated. The average number of water molecules within 4 Å distance from each residue was calculated along with respective standard errors (Fig. 3a,b, Table S3). It was observed that all the hydrophobic (non-polar) residues have consistently higher solvent exposure in the GTP bound state, whereas the hydrophilic (polar/charged) groups have higher exposure in the inactive, GDP bound state, suggesting that the SWI region becomes more hydrophobic in the GTP bound active state which could further facilitate effector recognition, as seen in the case of RhoA- AKAP-Lbc (Rho GEF)^57^ and Ras p21-Raf interactions^58^.

In order to understand the possible biological significance of the hydrophobic residues in the SWI region, we have presented sequence alignment comparison between the SWI region in H-Ras and RhoA (Fig S5). As compared with H-Ras, the SWI region in RhoA exhibits substitution by multiple non-polar residues in place of the charged residues in H-Ras at positions 33,35 and 39 in accordance with RhoA numbering. These substitutions should result in significantly different solvation behaviour of the solvent exposed region between Ras and Rho GTPases. Prior experimental studies highlight the flipping out of hydrophobic residues Val35, Val38, and Phe39 towards the solvent region in the GTP bound form^53^. The importance of hydrophobic contacts of switch I and II regions in protein-protein interaction has been illustrated by Dvorsky *et al*.^59^. It is interesting to note that the Glu37 (H-Ras Numbering) in H-Ras is substituted by Phe39 (RhoA Numbering) in RhoA, which is also found to be in significant correlation with Tyr34, as demonstrated earlier. Furthermore, comparison of structures of Ras (PDB: 1HE8) and Rho (PDB: 1CXZ) bound to their effectors show significant differences in terms of length of the SWI loop and replacement of Phe39 with Glu37. In Ras GTPases the length of the SWI loop is shorter by almost 12 residues (Fig. S5), compared to its Rho homolog. Also, Glu37 (in H-Ras) provides additional rigidity to the SWI region through either inter molecular or through intra-molecular interactions (Fig. S6). Thus, these stabilizing factors might make the Ras amiable to study the intermediate conformations SWI region using ensemble based experimental techniques. However, for Rho GTPases these additional stabilizing factors are unfavourable for capturing the state 1 like conformations. Therefore, biochemical and structural studies of mutants that drive the Rho GTPases towards state 1 conformation could further augment the current observations.

This observation poses two new questions: how does a conformational state with higher solvent exposure of hydrophobic residues can be stabilized in the GTP bound state and why state 1 like conformations are unfavourable to be detected with ensemble based experimental techniques, such as NMR? Although the stabilization might come from the hydrogen bonding interaction of a few residues (Tyr34 and Thr37) with the γ-phosphate of GTP, it is important to obtain a more quantitative picture. In order to understand the relative role of stabilization of the SWI conformations due to specific interactions, the net total energy of the loop between GDP and GTP bound form was computed and the difference of each contribution is shown in Fig. 3c. We have also decomposed the difference in total energy into individual contributions of the protein, solvent, nucleotide and the Mg^2+^ ion.

These calculations demonstrate the fine energetic interplay that leads to preferential stabilization of some conformational states over other depending on the nucleotide. In particular, the interaction with nucleotide and solvent seems to be the most important factor that affects the equilibrium (Fig. S7). In the GDP bound inactive state all the hydrophobic groups are buried and the hydrophilic groups are exposed. Thus this conformational state is highly favoured by the solvation energy (interaction with water). In case of the GTP bound active state the interaction with water becomes highly unfavourable (exposed hydrophobic and buried hydrophilic groups), but even higher favourable interaction with the nucleotide (GTP) counterbalances the solvation energy term to make the total energy lower for this conformation. This fine balance (energetic see-saw) is quite remarkably maintained across all the residues as well^60^. The residue-wise distribution of the interaction energies has been shown in the Supporting Information (Fig. S8).

The differential stability between the state 1 and state 2 conformations in the GTP bound state may also be explained based on the above energetic considerations. Based on the representative structures from free energy landscape, one can speculate that the state 1 conformation exhibits lack of stabilization due to the reduced favourable interaction with the nucleotide. Furthermore, the energetic balance between the penalty of exposing the hydrophobic residues, and the favourable hydrogen bonded interactions of GTP with Tyr34 and Thr37 would dictate the thermodynamic stability and population of these conformational substrates.

## Conclusions

We have presented here one of the very few fully atomistic MD simulation for the Rho GTPases. Using an unsupervised machine learning analysis of the conformational ensemble obtained from the MD trajectory as well as free energy calculation, we have demonstrated that the SWI loop of the GTPases may exist in various metastable states. In the GTP bound form SWI loop has state 1 and state 2 like conformations. For the G14V like mutant, the GTPases shifts towards the state 1 like conformation. The intermediate conformational sub-states have been characterized with respect to the unique side-chain orientation, particularly with respect to the residues Tyr34 and Phe39, both of which undergo large movement between the active and inactive conformational states.

We have also demonstrated that the characteristic feature of GTP bound active conformational state could be the highly exposed hydrophobic groups and less exposed hydrophilic groups, whereas the opposite scenario could characterize GDP bound inactive state. The stabilization of the exposed hydrophobic groups in the active state occurs because of the highly favourable interactions with the GTP. Thus, the fine balance between the interaction between nucleotide and solvent leads to the shift in the conformational equilibrium depending on the nucleotide binding state. This observation that binding to GTP significantly pushes the conformational ensemble to exposing a hydrophobic patch around the SWI loop in the active state strongly suggests that hydrophobic interactions might play a dominant role in effector recognition and binding through this region. Thus the current study provides a framework for looking at GTP bound inactive state of Rho GTPases. However, further experimental studies involving measurement of affinities of the SWI mutants (especially Y34S, T37S/T37A and F39E) for the effectors is required to characterize this state. Perhaps, crystal structures of these mutants might throw light on the conformational signatures of these states.

## Methods

### Structure selection

The crystal structures of RhoA for GDP bound and GTP bound states were selected as input structures for MD simulation (PDB: 1FTN and 1A2B respectively). The wild type structure for GTP bound form was obtained by back mutating the V14G in the crystal structure (PDB: 1A2B) using Coot program^61^. In case of GTP bound form, the GTP analogue was converted to GTP. The unbound form of the RhoA GTPase was simulated too. The nucleotide freeform for RhoA was obtained from the complex with GEF of Dbl family (PDB: 1LB1). All the systems preserved the Mg^2+^ ion together with the coordinating water molecules.

### Molecular dynamics simulations

MD simulations were performed using GROMACS 4.6.5 package^62^. The CHARMM27 force field with CMAP corrections^63^ was used for the protein and the parameters for ligands namely GDP and GTP were obtained from SwissParam^64^, a web service that provides topologies and parameters for small organic molecules, compatible with the CHARMM all atoms force field, for use with the GROMACS software. The partial atomic charges for the GDP and GTP molecules are listed in Tables S4 and S5 in the supplementary material. All the structures were inserted into cubic box with explicit solvent described by the TIP3P water model^65^ and simulated with periodic boundary conditions. The box sizes were set to ensure a distance of at least 1 nm between the protein and the box boundaries. This results in a 7.78 nm wide box with ~14700 water molecules for the nucleotide freeform, 7.72 nm wide box with ~14600 water molecules for the GDP bound form and 7.8 nm wide box with ~14700 water molecules for the GTP bound form. The systems were found to be negatively charged, thus in order to neutralise the simulation systems, 7 Na^+^ ions in the freeform system, 8 Na^+^ ions in the GDP bound system and 9 Na^+^ ions in GTP bound system were added. The solvated proteins were energy minimised using a steepest descent algorithm. All the systems were equilibrated for 300 ns in NPT ensemble using a modified Berendsen thermostat^66^ at 300 K and Parrinello-Rahman barostat^67^ at 1 bar. Long-range electrostatic interactions were calculated with the particle mesh Ewald (PME) summation method^68^ with a grid spacing of 0.16 nm and fourth-order cubic interpolation. For short range electrostatics and van der Waals, a cut-off distance of 1 nm was used. All covalent bonds were constrained using the LINCS algorithm^69^. The distance between the Mg^2+^ ion and the hydroxyl oxygen in Thr17 of RhoA was restrained to preserve the interactions between Mg^2+^ ion and its coordinating atoms. The integration time step was set to 2fs. Two different types of simulations were performed, atomistic MD simulation and well-tempered metadynamics simulation. The systems were equilibrated for 150 ns. The atomistic MD simulations were further performed in two replicas for 300 ns with each frame being saved at every 2 ps. Only one replica (300 ns) was used for the analysis. The metadynamics simulations were carried out for 1 μs.

### Distance based RMSD Parameter

Distance based RMSD (DRMSD) measures the structural deviation with respect to a reference structure based on the distances between all the pairs of atoms. This approach alleviates the need of aligning to a reference structure as required in the usual position based RMSD. The DRMSD between any conformation at time t (X_t_) and the reference structure (X_ref_) can be measured as:


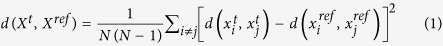


where N is the number of atoms and d(x_i_,x_j_) represents the distance between atoms i and j. We used the set of all C_α_ atoms of SWI loop for DRMSD calculation. Two reference structures (crystal structures of GDP and GTP-bound forms) were used to characterise the structural similarity/deviation with respect to the active and inactive states.

### Unsupervised Clustering

Unsupervised clustering was performed using the Weka software to cluster the conformations from the trajectories^70^. The k-means clustering method was carried out using the feature set consisting of the φ, ψ and χ dihedral angles of a subset of the evolutionary conserved residues of the SWI loop that undergo large changes in the dihedral angles between the active and inactive states (Table S6 and S7). The total number of output clusters was limited to four.

### Metadynamics simulations

We also carried out well-tempered Metadynamics^71^ to accelerate the conformational sampling of the Switch I region and to obtain the free energy landscape corresponding to the different nucleotide bound states. We used the Plumed code (ver. 2.0.2)^72^ to incorporate the Metadynamics functionalities in Gromacs. We used a “dihedral similarity” (*S*) parameter based on the backbone dihedral angles (φ/ψ) as the collective variable to distinguish the various conformational states of the Switch I region as defined below.


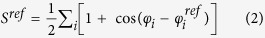


where the sum runs over the φ and ψ dihedral angles for all the residues of the Switch I region. The reference values (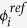) were taken from the respective angles in the crystal structure. The width of the Gaussian (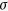) was set to 2.3 degrees for both the CVs with the initial height of Gaussians W = 0.1 kcal/mol added every 2 ps.

### Interaction energy calculation

The interaction energy for different components for the system, i.e. protein, water, Switch I region and Mg^2+^ ion was calculated using gromacs inbuilt tool g_energy. The total interaction energy is calculated as the sum of the electrostatic columbic interaction energy and Van der Waals interaction energy terms. The cutoff radius was set to 1 nm. All the atoms within the cutoff were included for the energy calculation.

## Additional Information

**How to cite this article:** Kumawat, A. *et al*. Nucleotide Dependent Switching in Rho GTPase: Conformational Heterogeneity and Competing Molecular Interactions. *Sci. Rep.* **7**, 45829; doi: 10.1038/srep45829 (2017).

**Publisher's note:** Springer Nature remains neutral with regard to jurisdictional claims in published maps and institutional affiliations.

## Supplementary Material

Supplementary Information
