# Supplementary material for: Nucleotide Dependent Switching in Rho GTPase: Conformational Heterogeneity and Competing Molecular Interactions
Source: Sci Rep. 2017 Apr 4;7:45829. doi: 10.1038/srep45829 (PMC5379185; doi:10.1038/srep45829)
Supplement: Supplementary Information [file srep45829-s1.pdf]

Supporting Information

Nucleotide Dependent Switching in Rho GTPases: Conformational Heterogeneity and Competing Molecular Interactions

Amit Kumawat,[‡] Suman Chakrabarty,[‡,*] and Kiran Kulkarni[†,*]

[‡]Physical and Materials Chemistry Division, CSIR-National Chemical Laboratory, Pune 411008, India; and [†]Division of Biochemical Sciences, CSIR-National Chemical Laboratory, Pune 411008, India

[*]Correspondence: s.chakrabarty@ncl.res.in, ka.kulkarni@ncl.res.in

Table S1. Summary of available Crystal structures of RhoA, Cdc42, Rac1 of (a) GDP bound form and (b) GTP bound form. # represents structures which are shown as overlaid on free energy surface in Fig. 3.

(a)

S.No.	PDB	Title
1	1CC0	Crystal structure of the RhoA.GDP-RhoGDI complex
2	1DPF	Crystal structure of a Mg-free form of RhoA complexed with GDP
3	1OW3	Crystal structure of RhoA.GDP.MgF_3^- in complex with RhoGAP
4	3LW8	Shigella IPGB2 in complex with human RhoA, GDP and Mg^{2+} (complex A)
5	3MSX	Crystal structure of RhoA.GDP.MgF_3^- in complex with GAP domain of ARHGAP20
6	1DS6	Crystal structure of a Rac-RhoGDI complex
7	1G4U	Crystal structure of the salmonella tyrosine phosphatase and GTPase activating protein sptp bound to Rac1
8	1HE1	Crystal structure of the complex between the GAP domain of the pseudomonas aeruginosa exos toxin and human Rac
9	1HH4	Rac1-RhoGDI complex involved in NADPH oxidase activation
10	1I4D	Crystal structure analysis of Rac1-GDP complexed with arfaptin(p21)
**11#**	**2G0N**	**The crystal structure of the human Rac3 in complex with GDP and chloride**
12	2H7V	Co-crystal structure of YPKA-Rac1
**13#**	**2W2T**	**Rac2 (G12V) in complex with GDP**
**14#**	**1A4R**	**G12V mutant of human placental Cdc42 GTPase in the GDP form**
**15#**	**1AN0**	**Cdc42Hs-GDP complex**
16	1GRN	Crystal structure of the Cdc42/Cdc42GAP/AlF_3 complex.
17	4DID	Crystal structure of salmonella effector N-terminal domain SOPB in complex with Cdc42

(b)

S.No.	PDB	Title
18	1CXZ	Crystal structure of human RhoA complexed with the effector domain of the protein kinase PKN/PRK1
**19#**	**1KMQ**	**Crystal structure of a constitutively activated RhoA mutant (q63l)**
20	1Z2C	Crystal structure of MDIA1 GBD-FH3 in complex with RhoC-GMPPNP
**21#**	**2GCP**	**Crystal structure of the human RhoC-GSP complex**
22	3KZ1	Crystal structure of the complex of PDZ-RhoGEF DH/PH domains with GTP-γ-s activated RhoA
**23#**	**3TVD**	**Crystal structure of mouse RhoA-GTP complex**
24	4F38	Crystal structure of geranylgeranylated RhoA in complex with RhoGDI

		in its active GPPNHP-bound form
25	1E96	Structure of the Rac/p67phox complex
26	1I4T	Crystal structure analysis of Rac1-GMPPNP in complex with arfaptin
27	1MH1	Small G-protein complexed with guanosine-5'-(β,γ-imido) triphosphate (GPPNP)
28	2FJU	Activated Rac1 bound to its effector phospholipase C β2
29	3RYT	The Plexin A1 intracellular region in complex with Rac1
**30[#]**	**3SBD**	**Crystal structure of Rac1 P29S mutant**
**31[#]**	**3SUA**	**Crystal structure of the intracellular domain of Plexin-B1 in complex with Rac1**
**32[#]**	**3TH5**	**Crystal structure of wild-type Rac1**
**33[#]**	**4GZM**	**Crystal structure of Rac1 F28L mutant**
34	1NF3	Structure of Cdc42 in a complex with the GTPase-binding domain of the cell polarity protein, par6
35	3EG5	Crystal structure of MDIA1-TSH GBD-FH3 in complex with Cdc42-GMPPNP
36	1CF4	Cdc42/ACK GTPase-binding domain complex

Table S2. Relative free energy of the conformational states and the barrier between different Minima on Free Energy Surface in nucleotide unbound and bound states.

S.No.	System	Transition States	Free Energy (kcal)
1	Nucleotide freeform	ΔG (A)	0
		ΔG (C)	4.6
		$\Delta G^{\ddagger}$ (A→C)	7
2	GDP bound state	ΔG (A)	0
		ΔG (B)	0.5
		$\Delta G^{\ddagger}$ (A→B)	2
3	GTP bound state		
	(a) Wild Type	ΔG (A)	0
		ΔG (B)	0.7
		$\Delta G^{\ddagger}$ (A→B)	5
	(b) Mutant G14V	ΔG (A)	0
		ΔG (B)	-0.4
		$\Delta G^{\ddagger}$ (A→B)	2

Table S3: Standard error values for average number of water molecules around polar and non-polar residues of Switch I region (28-40) in GDP bound and GTP bound form.

Residue Number	Error (GDP)	Error (GTP)
28	0.15	0.14
29	0.24	0.06
30	0.07	0.18
31	0.07	0.09
32	0.09	0.26
33	0.13	0.11
34	0.21	0.30
35	0.16	0.05
36	0.04	0.26
37	0.20	0.18
38	0.25	0.09
39	0.17	0.09
40	0.30	0.17

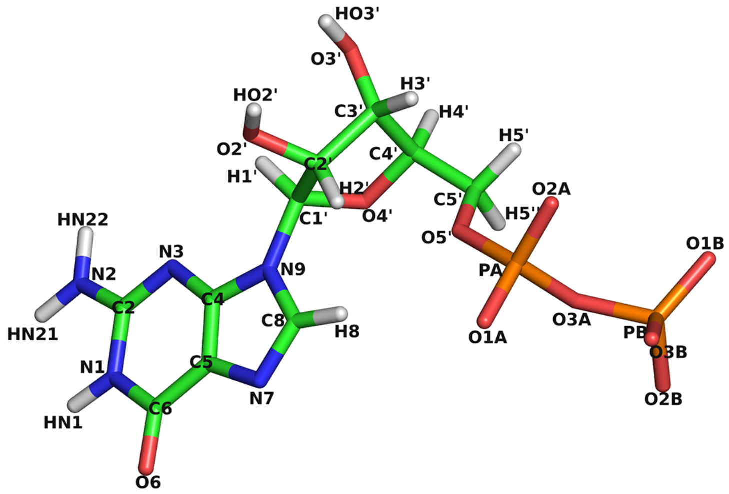

Table S4. Description of partial atomic charges for GDP molecule derived from SwissParam web service. Atom names are described in the figure above.

Atom Number	Atom Name	Charge
1	N9	0.0476
2	C8	0.0365
3	N7	-0.5653
4	C5	0.1412
5	C6	0.716
6	N1	-0.49
7	C2	0.56
8	N3	-0.576
9	C4	-0.0256
10	PB	1.3712
11	O1B	-1.0333
12	O2B	-1.0333
13	O3B	-1.0333
14	O3A	-0.5424
15	PA	1.4424
16	O1A	-0.95
17	O2A	-0.95
18	O5'	-0.5512
19	C5'	0.28
20	C4'	0.28
21	O4'	-0.56
22	C3'	0.28
23	O3'	-0.68

Atom Number	Atom Name	Charge
24	C2'	0.28
25	O2'	-0.68
26	C1'	0.5356
27	O6	-0.57
28	N2	-0.85
29	H5'	0
30	H5''	0
31	H4'	0
32	H3'	0
33	H2'	0
34	H1'	0
35	H8	0.15
36	HN1	0.37
37	HN21	0.4
38	HN22	0.4
39	HO3'	0.4
40	HO2'	0.3999

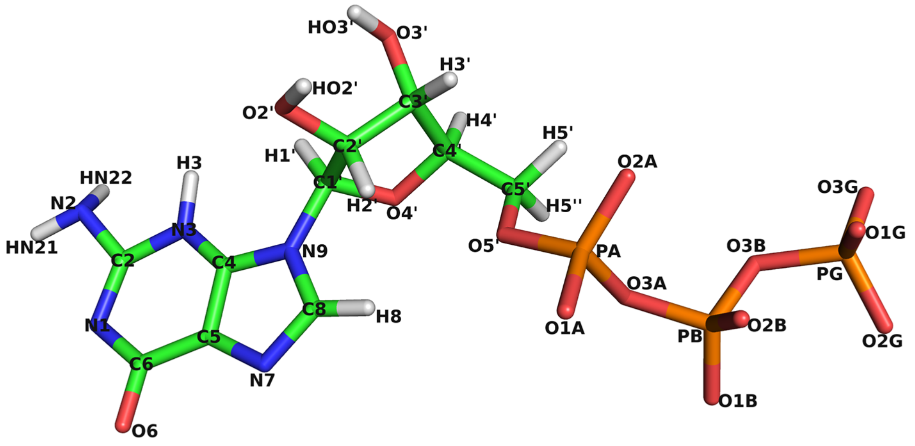

Table S5. Description of partial atomic charges for GTP molecule derived from SwissParam web service. Atom names are described in the figure above.

Atom Number	Atom Name	Charge
1	C6	0.867
2	N1	-0.661
3	C2	0.55
4	N3	-0.534
5	C4	-0.0676
6	C5	0.1412
7	N7	-0.5653
8	C8	0.0365
9	N9	0.0476
10	O6	-0.57
11	N2	-0.85
12	C1'	0.5356
13	O4'	-0.56
14	C2'	0.28
15	O2'	-0.68
16	C3'	0.28
17	O3'	-0.68
18	C4'	0.28
19	C5'	0.28
20	O5'	-0.5512
21	PA	1.4424
22	O1A	-0.95
23	O2A	-0.95

Atom Number	Atom Name	Charge
24	O3A	-0.5424
25	PB	1.4424
26	O1B	-0.95
27	O2B	-0.95
28	O3B	-0.5424
29	PG	1.3712
30	O1G	-1.0333
31	O2G	-1.0333
32	O3G	-1.0333
33	H3	0.4
34	H8	0.15
35	H1'	0
36	H2'	0
37	H3'	0
38	H4'	0
39	H5'	0
40	H5''	0
41	HN21	0.4
42	HN22	0.4
43	HO3'	0.4
44	HO2'	0.3999

Table S6. Selection criteria for residues used in unsupervised clustering. The residues for machine learning where selected on the basis of the difference in the torsional angles from the GDP and GTP bound equilibrated structure ($\Delta X = X_{GDP} - X_{GTP}$). The angles included for the analysis include φ, ψ, χ1, χ2. All those residues with major change in the orientation of the backbone/side chain (highlighted in red) were selected for k-means clustering.

Switch I	φ_{GDP}	φ_{GTP}	$\Delta\varphi$	ψ_{GDP}	ψ_{GTP}	$\Delta\psi$	$\chi1_{GDP}$	$\chi1_{GTP}$	$\Delta\chi1$	$\chi2_{GDP}$	$\chi2_{GTP}$	$\Delta\chi2$
Asp28	65.5	67.4	-1.9	38.7	40.0	-1.3						
Gln29	-161.0	-128.3	-32.6	153.4	141.9	11.5						
**Phe30**	-78.7	-108.7	30.1	136.4	125.0	11.4	-171.6	-174.1	2.6	-102.0	63.3	**-165.3**
Pro31	-102.6	-53.2	-49.4	107.7	148.6	-40.9						
Glu32	-66.0	-74.0	8.0	-30.5	-36.4	5.9						
Val33	-138.9	-152.5	13.6	157.0	161.7	-4.8						
**Tyr34**	68.3	-74.0	**142.3**	41.1	124.0	-82.9	-47.4	179.7	**-227.2**	75.1	26.9	48.2
Val35	-70.7	-110.7	40.0	119.9	128.9	-9.0						
**Pro36**	-64.6	-53.5	-11.1	162.6	122.9	39.6	47.2	-31.1	**78.2**	-22.9	-26.1	3.2
**Thr37**	-58.4	-73.9	15.5	-47.9	128.2	**-176.2**	-51.4	-50.6	-0.8			
**Val38**	-147.1	-86.9	-60.1	170.5	-54.8	**225.3**	-64.0	-169.4	**105.4**			
**Phe39**	-127.3	-144.6	17.3	134.4	150.3	-16.0	-154.5	52.2	**-206.7**	-104.1	-92.9	-11.2
Glu40	-156.9	-139.3	-17.6	155.6	146.5	9.0						

Table S7. List of residues used for k-means clustering. Selection of residues is based on the difference in the dihedral angles in the equilibrated GDP bound and GTP bound form as highlighted in Table S1.

Sr. No	Switch I Residues	Dihedral Angles
1	PHE30	$\varphi, \psi, \chi_1, \chi_2$
2	TYR34	$\varphi, \psi, \chi_1, \chi_2$
3	PRO36	$\varphi, \psi, \chi_1, \chi_2$
4	THR37	φ, ψ, χ_1
5	VAL38	φ, ψ, χ_1
6	PHE39	$\varphi, \psi, \chi_1, \chi_2$

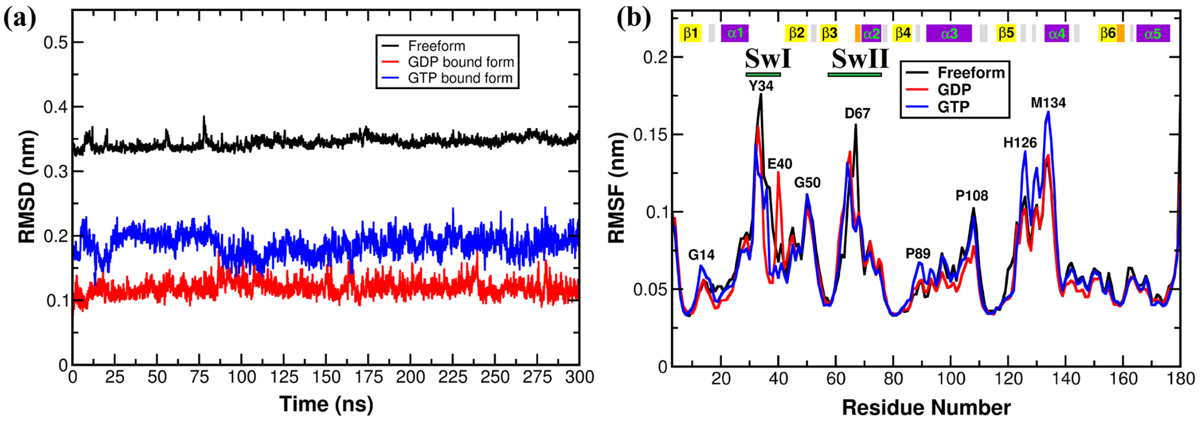

Figure S1. Root mean square deviation (RMSD) and fluctuations (RMSF) for the equilibrated part of the trajectory (300ns). (a) The trajectory was fitted and RMSD was calculated for the backbone atoms with respect to the respective crystal structures. It is evident from the plot that the systems are well equilibrated and there are no major secondary structural changes in the GTPase. (b) The RMSF profile exhibits average fluctuation per residue for the complete trajectory. The protein shows enhanced fluctuations in the SwI and SwII region in the freeform state. In the GTP bound form, increased fluctuations are observed in the insert helix towards the C-terminal.

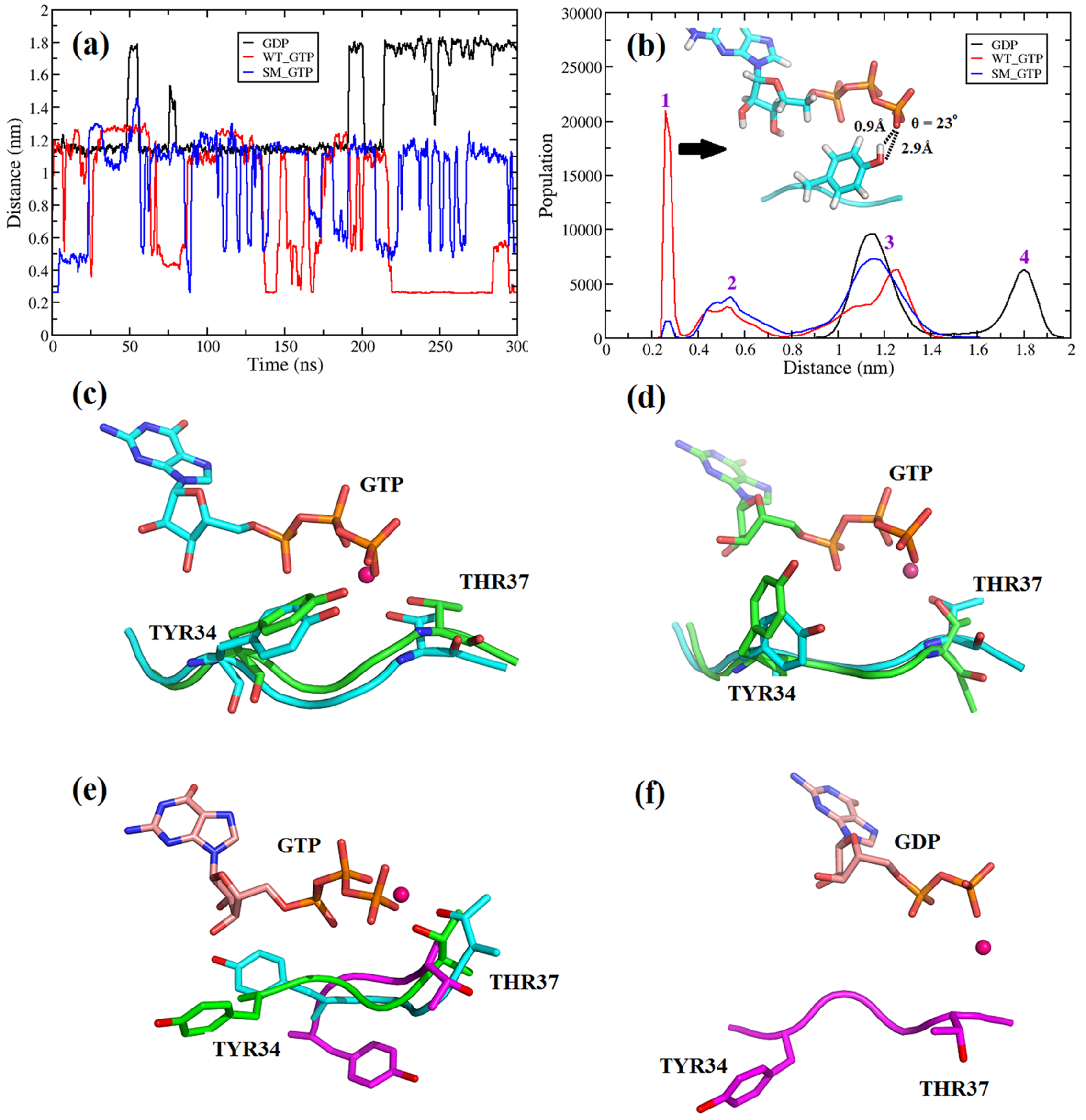

Figure S2. Structural states in different systems based on the Tyr34 orientation. (a) Time evolution and (b) probability distribution of minimum distance between the oxygen atom of hydroxyl group in Tyr34 and oxygen atoms of terminal phosphate group of GDP and GTP molecule in GDP bound form, wild type GTP bound form (WT_GTP), single mutation G14V-GTP bound form (SM_GTP). In case of GDP bound form, oxygen atoms of β-phosphate group are considered where as in GTP bound form, γ-phosphate oxygen atoms are considered for minimum distance calculation. The peak labelled as 1 represents the existence of hydrogen bond between the oxygen atom of hydroxyl group in Tyr34 and the terminal phosphate. A representative image for the orientation of Tyr34 sidechain in GTP bound form is shown as inset image. The details of the hydrogen bond formation are also shown in the figure (Bond distance= 2.9Å, angle = 23°). The existence of multiple states is evident from

the probability distribution plot which also exhibits a distinct nucleotide dependent shift of population. (c - f) Superimposition images of structures from each of the labelled four states coloured as WT-GTP (cyan), G14V-GTP (green), and GDP (magenta).

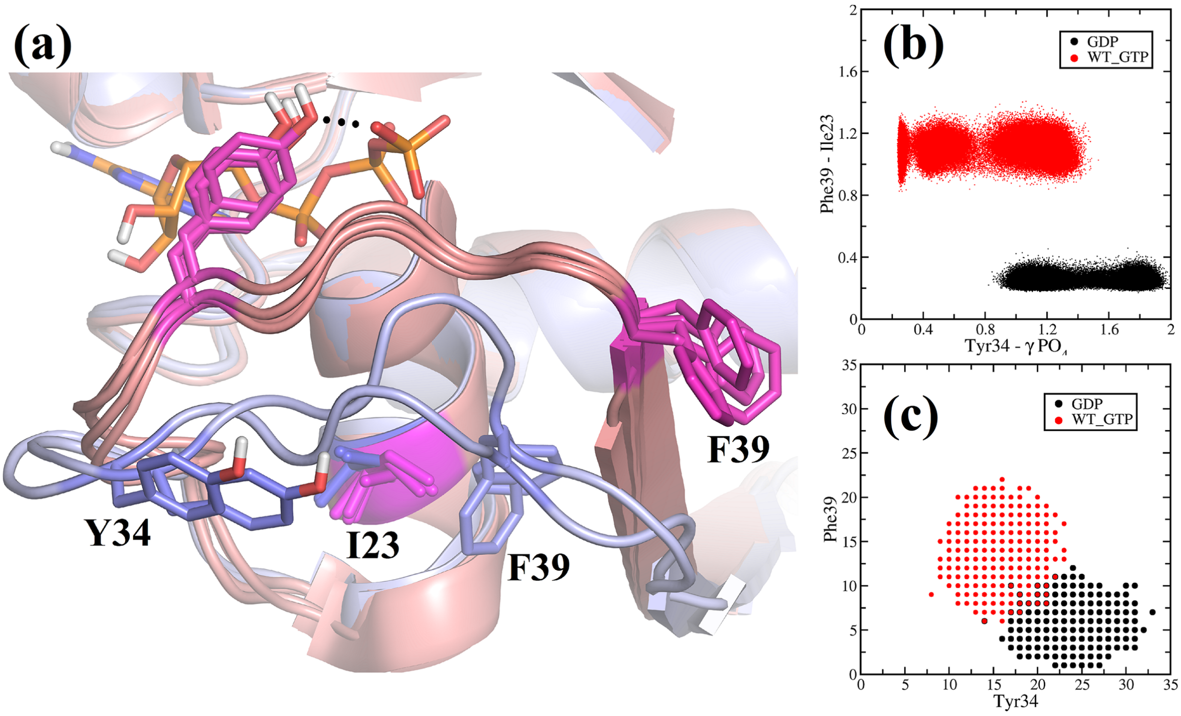

Figure S3: (a) Orientation of Phe39 and Tyr34 in GDP bound crystal structures (Blue) and GTP bound crystal structures (Pink). The nearest possible interaction is also shown in the figure. In case of GTP bound form, Tyr34 sidechain forms hydrogen bond with the γ-phosphate oxygen atom whereas in case of GDP bound form, Phe39 sidechain interacts with Ile23 sidechain. Both the interactions exhibits antagonistic behaviour where one breaks and other is formed and vice versa. (b) Minimum distance between the nearest possible interaction. (c) Solvent exposure of Tyr34 and Phe39 sidechain. Average number of water molecules was calculated around the Tyr34 and Phe39 residues. It is observed that Tyr34 is more solvent exposed in GDP bound form as compared to the GTP bound form, whereas Phe39 is more solvent exposed in the GTP bound form as compared to the GDP bound form. The 2D plot corroborates our claim that this two residues show distinct sidechain orientation in different nucleotide bound state and there exists a correlation among their orientation.

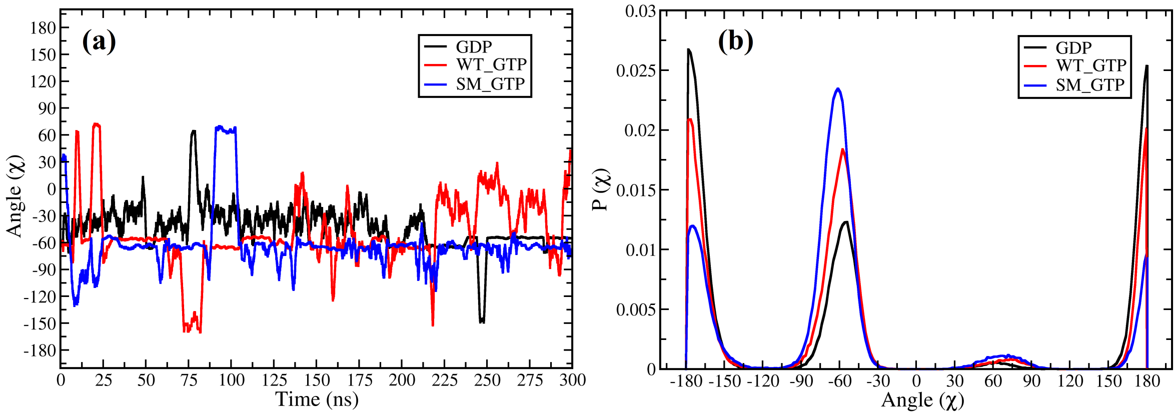

Figure S4. Frequency of Tyr34 sidechain orientation. (a) Time evolution and probability distribution of χ dihedral angle of Tyr34.

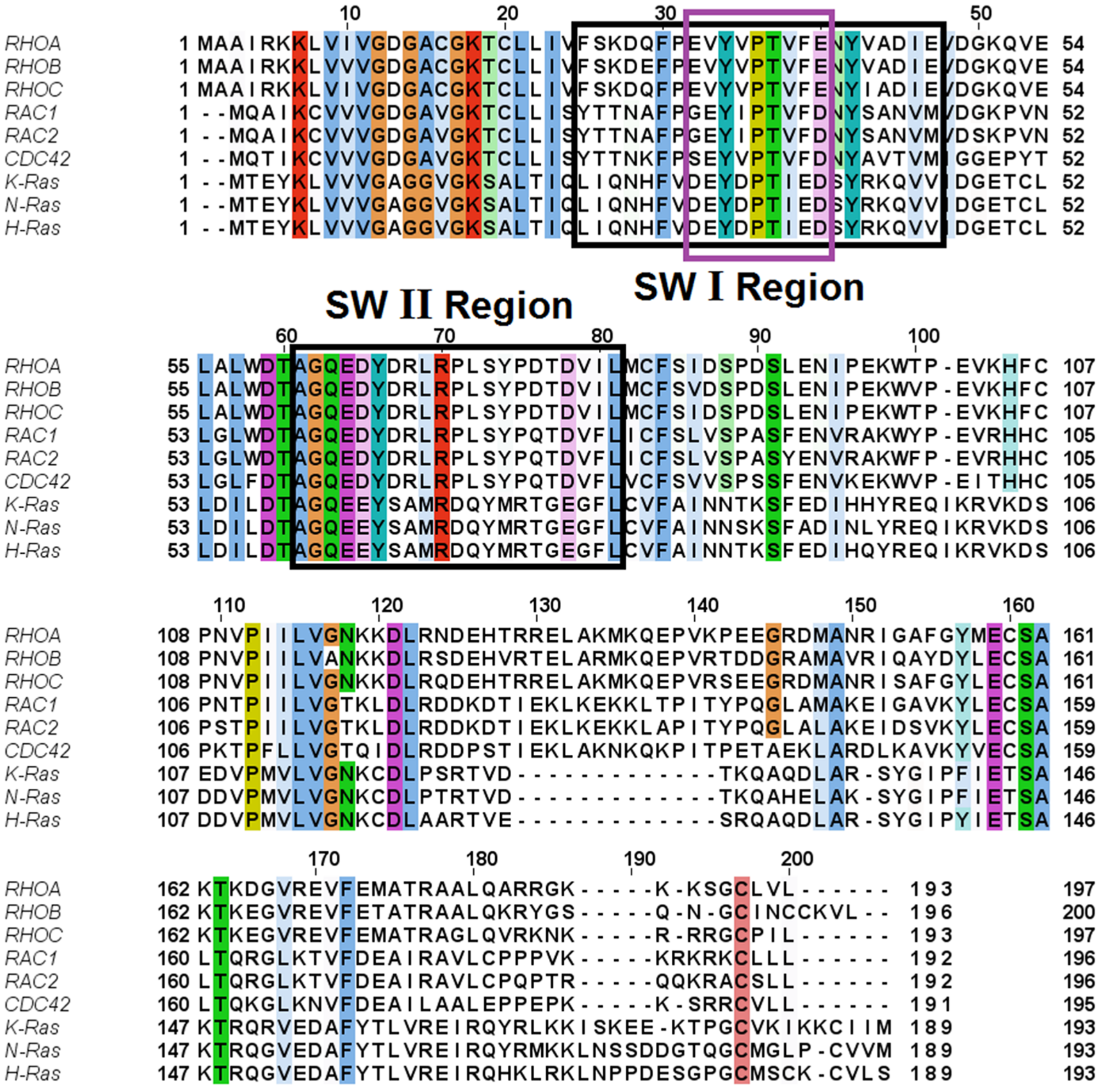

Figure S5. Multiple sequence Alignment of human Rho GTPases using Clustal-Ω. Residues with greater than 65% identity/conservation are highlighted using Clustal X colouring scheme. The accession numbers are P06749 (RhoA), P01121 (RhoB), P08134 (RhoC), P15154 (Rac1), P15153 (Rac2), P25763 (Cdc42), P01116 (K-Ras), P01111 (N-Ras), and P01112 (H-Ras). The switch regions in Rho and Ras are highlighted in black and violet colour respectively.

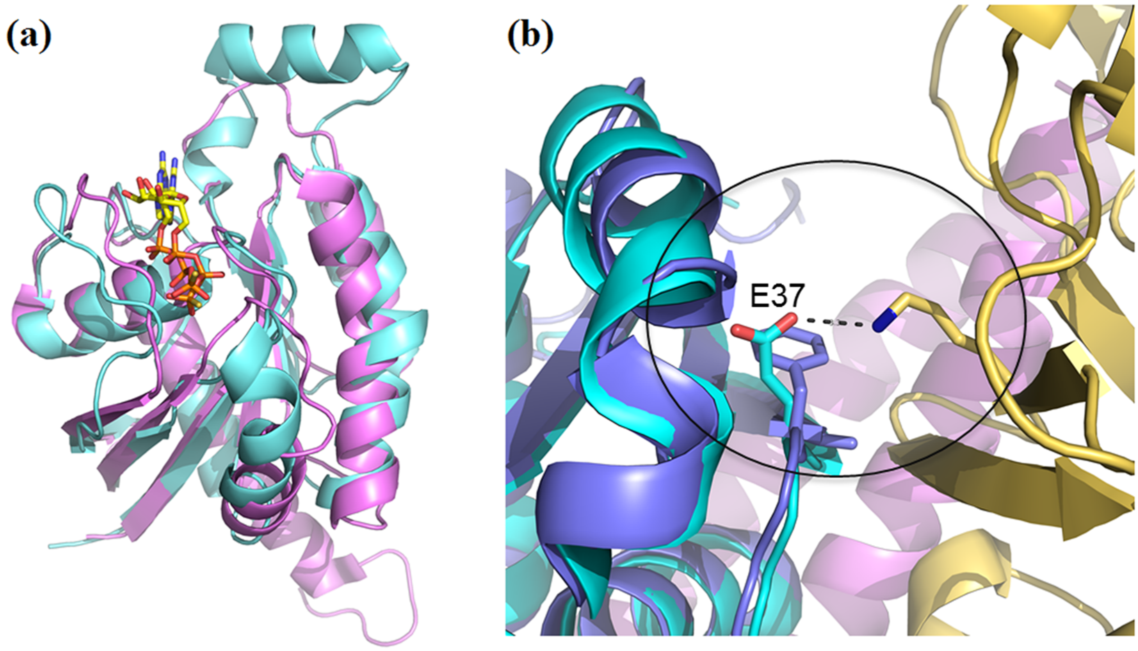

Figure S6. Structural comparison of H-Ras and RhoA protein in their effector bound state. (PDB: 1HE8 (H-Ras) and 1CXZ (RhoA))**.** (a) The figure highlights the difference in SWI loop region between H-Ras (magenta) and RhoA (cyan). (b) Molecular picture of effector recognition between K-Ras (cyan) and RhoA (blue). Superimposition of effector bound states in K-Ras and RhoA highlights the importance of substitution of Glu37 in K-Ras (RhoA numbering) interaction with Ras effector (yellow) eventually increasing the rigidity of the SWI region.

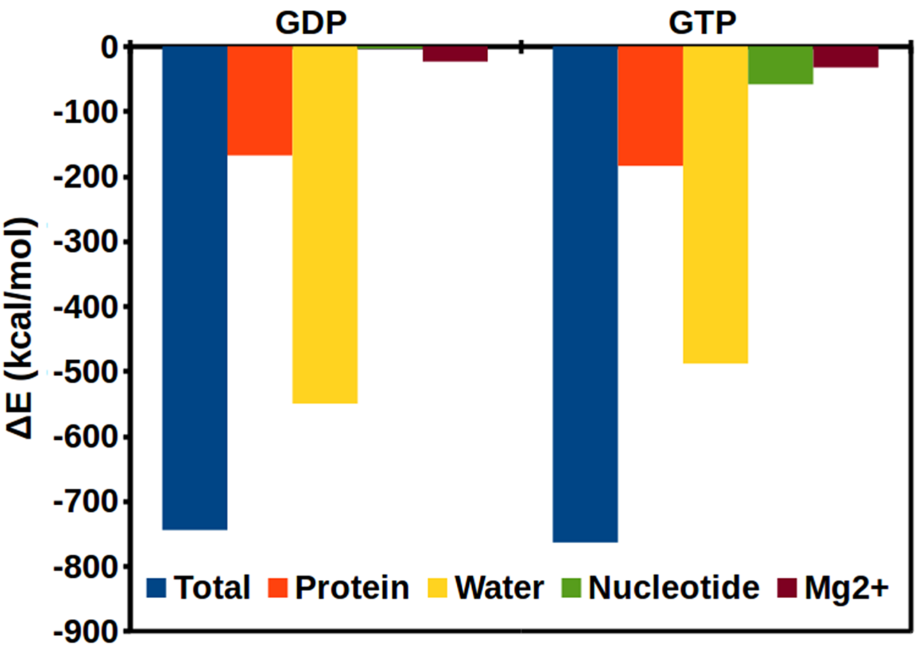

Figure S7. Interaction energy distribution for GDP and GTP bound state of switch I region. It can be observed that the loss in the interaction energy in the GTP bound form due to the increase in solvent exposure of the switch I region is compensated by the energy contribution due to nucleotide.

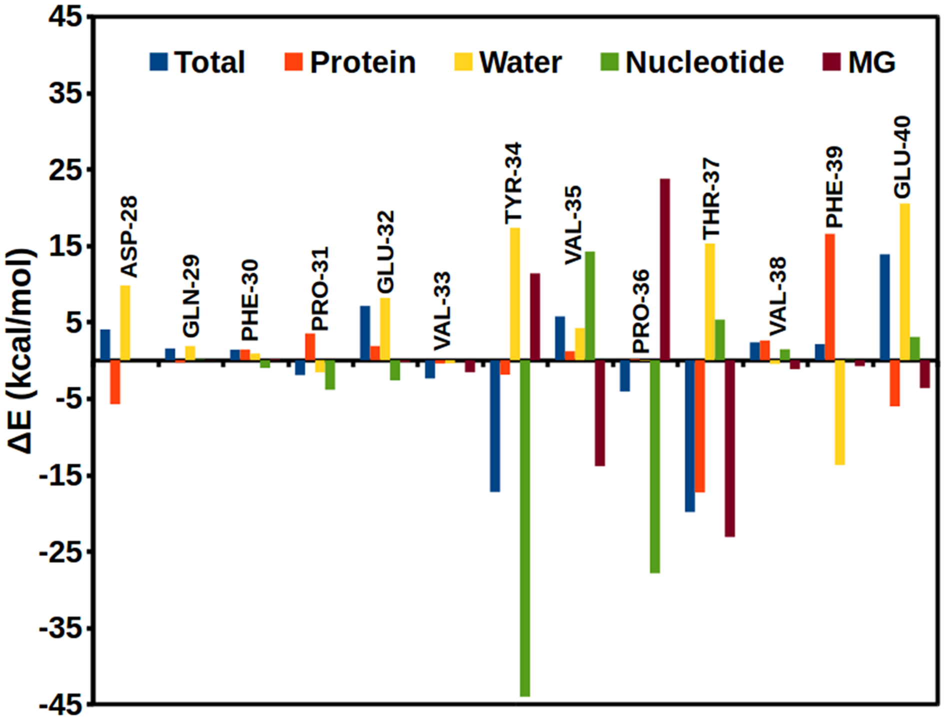

Figure S8. Residue wise distribution of net interaction energy of switch I region between the two states, $\Delta E = E_{GTP} - E_{GDP}$, dissected into different components i.e. total energy (blue), protein (orange), water (yellow), nucleotide (green) and the Mg^{2+} ion (brown). The energetic balance between different components can be observed among SWI loop residues.
